# Development of Bivalent Aptamer-DNA Carrier-Doxorubicin Conjugates for Targeted Killing of Esophageal Squamous Cell Carcinoma Cells

**DOI:** 10.3390/ijms25147959

**Published:** 2024-07-21

**Authors:** Tianlu Zhang, Kai Yin, Xidong Niu, Xue Bai, Zhaoting Wang, Mengmeng Ji, Baoyin Yuan

**Affiliations:** 1 School of Basic Medical Sciences, Zhengzhou University, Zhengzhou 450001, China; tianlu09062023@163.com (T.Z.); yink2000@163.com (K.Y.); nxd20031118@163.com (X.N.); baixue739808@163.com (X.B.); wangzhaoting7@163.com (Z.W.); jimeng0203@163.com (M.J.); 2 Henan Provincial Cooperative Innovation Center for Cancer Chemoprevention, Zhengzhou 450001, China; 3 State Key Laboratory of Esophageal Cancer Prevention & Treatment, Zhengzhou University, Zhengzhou 450001, China

**Keywords:** ESCC, bivalent aptamer, DNA carrier, Dox, targeted chemotherapy

## Abstract

Esophageal cancer ranks the seventh in cancer incidence and the sixth in cancer death. Esophageal squamous cell carcinoma (ESCC) accounts for approximately 90% of the total cases of esophageal cancer. Chemotherapy is the most effective drug-based method for treatment of esophageal cancer. However, severe side effects of traditional chemotherapy limit its treatment efficacy. Targeted chemotherapy can deliver chemotherapeutic drugs to cancer cells and specifically kill these cells with reduced side effects. In the work, the bivalent aptamer-DNA carrier (BAD) was designed by using an ESCC cell-specific aptamer as the recognition molecule and a GC base-rich DNA sequence as the drug carrier. With doxorubicin (Dox) as chemotherapeutic drugs, the bivalent aptamer-DNA-Dox conjugate (BADD) was constructed for targeted killing of ESCC cells. Firstly, the truncated A2(35) aptamer with a retained binding ability was obtained through optimization of an intact A2(80) aptamer and was used to fuse with DNA carrier sequences for constructing the BAD through simple DNA hybridization. The results of gel electrophoresis and flow cytometry analysis showed that the BAD was successfully constructed and had a stronger binding affinity than monovalent A2(35). Then, the BAD was loaded with Dox drugs to construct the BADD through noncovalent intercalation. The results of fluorescence spectra and flow cytometry assays showed that the BADD was successfully constructed and can bind to target cells strongly. Confocal imaging further displayed that the BADD can be specifically internalized into target cells and release Dox. The results of CCK-8 assays, Calcein AM/PI staining, and wound healing assays demonstrated that the BADD can specifically kill target cells, but not control cells. Our results demonstrate that the developed BADD can specifically deliver doxorubicin to target ESCC cells and selectively kill these cells, offering a potentially effective strategy for targeted chemotherapy of ESCC.

## 1. Introduction

Esophageal cancer is the seventh most common cancer and the sixth most common cause of cancer death worldwide [1]. Esophageal cancer mainly includes two subtypes: esophageal squamous cell carcinoma (ESCC) and esophageal adenocarcinoma (EAC). ESCC accounts for approximately 90% of esophageal cancer cases [2,3]. The major clinical treatment methods of esophageal cancer are surgical resection, radiotherapy, and chemotherapy [4,5]. Due to lack of widely overexpressed molecular targets, molecular-targeted therapy for esophageal cancer using small molecular inhibitors and monoclonal antibodies is not ideal [6]. Additionally, reliable specific recognition molecules for ESCC are lacked, which makes targeted delivery of drugs for ESCC difficult. Although immunotherapy is popular in recent years, it is limited in treatment of esophageal cancer because of low remission rates [7]. Traditional chemotherapy remains the most effective drug-based treatment for esophageal cancer, despite its severe side effects.

Targeted chemotherapy can specifically kill cancer cells by delivering chemotherapeutic drugs to target cancer cells but not normal cells, thereby reducing side effects and improving treatment efficacy of chemotherapeutic drugs. Doxorubicin (Dox) is an FDA-approved broad-spectrum chemotherapeutic drug to treat various cancers, including esophageal cancer [8,9]. Dox can nonspecifically enter into normal cells and lead to severe side effects, especially cardiotoxicity, which heavily restricts its clinical application [10,11,12]. It is a good way to resolve this issue to specifically deliver Dox to cancer cells for targeted chemotherapy of cancers. Because Dox can noncovalently intercalate into GC base-pairs of double-stranded DNA, DNA structures can be used as an ideal drug carrier for targeted delivery of Dox to cancer cells [13,14,15].

Aptamers are short single-stranded DNA or RNA molecules that can specifically recognize targets with high affinity [16]. Aptamers are usually selected through the systematic evolution of ligands by the exponential enrichment (SELEX) technique [17,18]. Known as novel recognition molecules, aptamers have various advantages over protein antibodies, such as easy synthesis, easy modification, flexible programmability, low cost, low immunogenicity, and low molecular weight [19,20]. Cancer cell-specific aptamers can be obtained via Cell-SELEX and are attracting more and more attention in cancer diagnosis, biomarker discovery, and targeted therapy [21,22,23,24,25,26]. For example, we have previously developed several aptamers target to ESCC cells and successfully identified potential cancer biomarkers using the aptamers [27,28,29]. Easy chemical modification and flexible design of aptamers allow them to carry chemotherapeutic drugs through covalent coupling or noncovalent intercalation and specifically deliver drugs to cancer cells via aptamer recognition, thus achieving targeted killing of cancer cells [30,31,32,33].

Bivalent aptamers have not only stronger binding ability and biostability than monovalent aptamers, but also a lower molecular weight than multivalent aptamers to penetrate tissues, which are more appropriate to use as recognition molecules for cancer cell targeting [34,35,36]. In addition, bivalent aptamers are facile to synthesize and construct and are controllable for drug loading. In previous work, we have developed an intact A2 aptamer that specifically binds to ESCC KYSE410 cells through Cell-SELEX and identified integrin β1 that is widely overexpressed in ESCC as the molecular target of A2 aptamer [27]. In this study, we aimed to obtain a truncated A2 aptamer by optimizing an intact A2 aptamer and then construct the bivalent aptamer-DNA carrier (BAD) through simple DNA hybridization by using the truncated A2 aptamer as the targeting element. The drug loading sequences (DNA carrier) located on the hybridization region of the BAD and were rich in GC base-pairs. With Dox as the chemotherapeutic drug, bivalent aptamer-DNA carrier-Dox conjugates (BADD) were constructed by noncovalently intercalating Dox into GC base-pairs of the DNA carrier. The BADD could specifically bind to target KYSE410 cells and then be internalized into target cells, killing target cells by releasing Dox. The proposed BADD may provide a simple and useful strategy for targeted chemotherapy of ESCC.

## 2. Results and Discussion

### 2.1. Design Principle of the BADD

**Figure 1:**
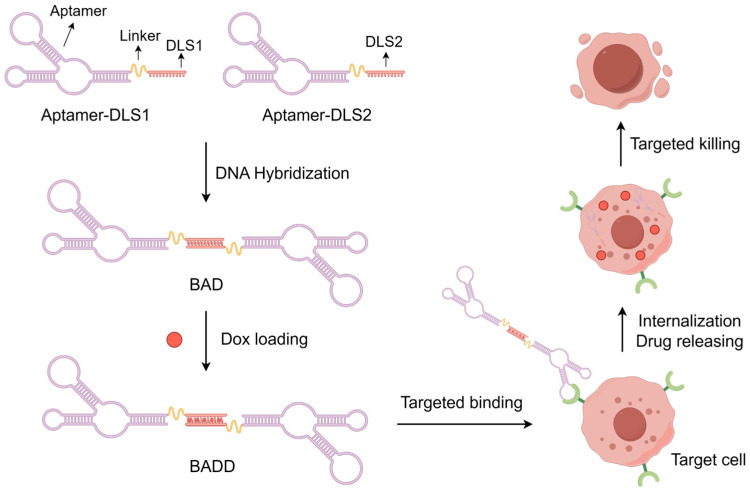
Schematic illustration of the design principle of the BADD for targeted killing of cancer cells.

To construct bivalent aptamer-DNA carrier-Dox conjugate (BADD), the bivalent aptamer-DNA carrier (BAD) was first designed. As shown in Figure 1, cancer cell-specific aptamer sequences are fused with drug loading sequences 1 (DLS1) and 2 (DLS2) separately, and the fused sequences are termed as Aptamer-DLS1 and Aptamer-DLS2, respectively. DLS1 and DLS2 are complementary to each other and rich in GC base-pairs. By mixing Aptamer-DLS1 with Aptamer-DLS2, the BAD can be simply constructed through base-pairing of DLS1 and DLS2. Doxorubicin (Dox) is a conventional FDA-approved chemotherapeutic drug to effectively treat various cancers. Due to its unique structure, Dox can noncovalently intercalate into GC base-pairs of double-stranded DNA sequences. Using this property, Dox molecules are loaded into the BAD that rich in GC base-pairs, which comprise the BADD. Because of the bivalent aptamer-induced specific and strong binding, the BADD can specifically recognize and enter into the target cells and release Dox for selective killing of cancer cells.

### 2.2. Optimization of the Aptamer

**Figure 2:**
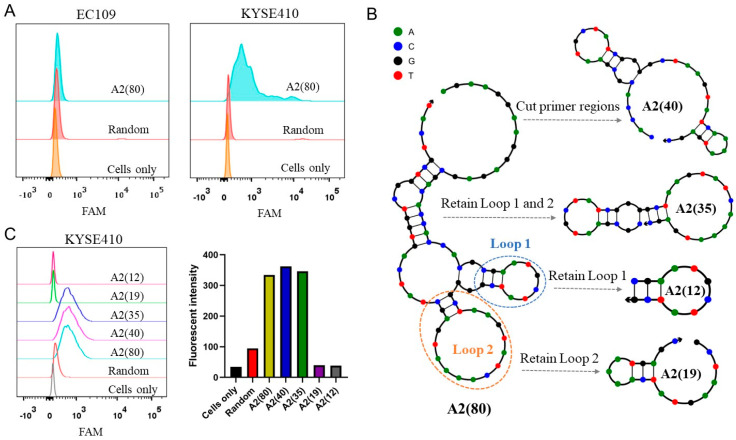
Optimization of the intact A2 aptamer. (**A**) Flow cytometry analysis of target KYSE410 and control EC109 cells incubated with the intact A2 aptamer. (**B**) Optimization strategies of the intact A2 aptamer. (**C**) Flow cytometry analysis of KYSE410 cells incubated with various truncated aptamers of A2. The corresponding quantitative fluorescent intensities were calculated.

In previous work, we have developed an intact A2(80) aptamer that can recognize ESCC cells, but not normal esophageal epithelial cells. The molecular target of A2(80) was identified as integrin β1 that is overexpressed in most ESCC cells, which possesses great potential for diagnosis and therapy of ESCC. Therefore, the A2(80) aptamer was used as a parental recognition molecule for construction of the BADD for targeted chemotherapy of ESCC. Because intact A2(80) is too long (80 bases) and contains redundant sequences that may affect its binding, we needed to optimize A2(80) to achieve a better truncated A2 aptamer. Flow cytometry analysis was used to detect the binding ability of A2(80) to target KYSE410 and control EC109 cells. The results showed that A2(80) had a good binding ability to target KYSE410 cells, but it almost did not bind to control EC109 cells (Figure 2A), verifying that A2 could specifically recognize target KYSE410 cells. The secondary structure of A2(80) was simulated by NUPACK software. As shown in Figure 2B, A2(80) includes two main loop structures, which probably contribute to aptamer binding according to previous reports [37,38,39]. Four truncated aptamer sequences were obtained by using four optimization methods (Figure 2B). A2(40) was obtained by removing the primer sequences at both the ends of A2(80). A2(35) was obtained by retaining both loops 1 and 2. A2(12) and A2(19) were obtained by retaining loops 1 and 2, respectively. The detailed aptamer sequences are listed in Table 1. These truncated sequences were labeled with a fluorescein amidite (FAM) fluorescent group for target cell binding assays by flow cytometry. As shown in Figure 2C, both A2(12) and A2(19) lost their binding abilities to target cells, while A2(40) and A2(35) retained a similar binding ability compared to A2(80), suggesting that both loops 1 and 2 are necessary in aptamer binding. Considering A2(35) has a good binding ability with a shorter length, it was selected for the subsequent experiments.

### 2.3. Construction of the BAD

**Figure 3:**
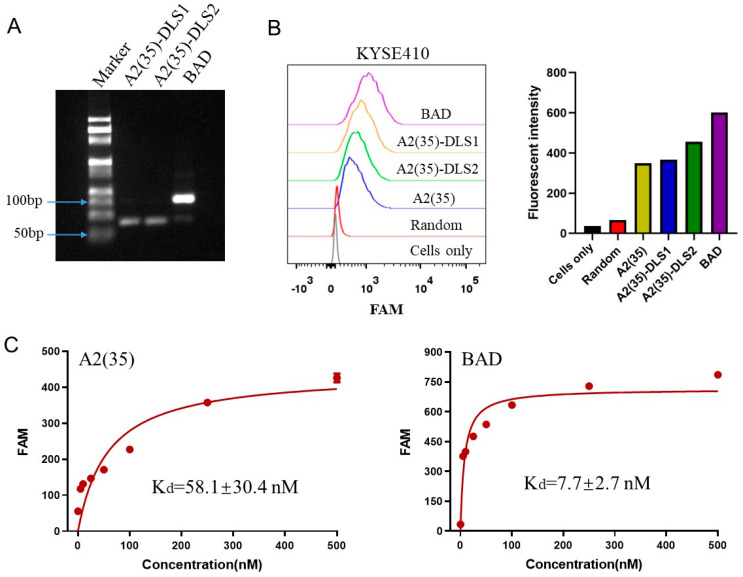
Construction and characterization of the BAD. (**A**) Formation of the BAD was analyzed by agarose gel electrophoresis. (**B**) Binding abilities of 250 nM of A2(35)-DLS1, A2(35)-DLS2, and the BAD to target KYSE410 cells detected by flow cytometry. Quantitative fluorescence intensity was calculated. (**C**) Determination of the dissociation constants (K_d_) of A2(35) and the BAD to target KYSE410 cells. Fluorescence intensity values were obtained by flow cytometry, and K_d_ was calculated by GraphPad Prism 8 software.

To construct the BAD, a truncated A2(35) aptamer was fused with GC-rich DLS1 and DLS2 sequences separately. The fused A2(35)-DLS1 and A2(35)-DLS2 sequences (Table 1) were mixed in equal proportions to form the BAD through simple base-pairing. The agarose gel electrophoresis experiment was performed to verify the construction of the BAD. As shown in Figure 3A, the molecular weight of the product formed by mixing A2(35)-DLS1 and A2(35)-DLS2 became significantly larger, indicating that A2(35)-DLS1 and A2(35)-DLS2 successfully hybridized to form the BAD.

### 2.4. Characterization of the BAD

After the successful construction of the BAD, flow cytometry was used to evaluate its binding ability to target cells. The results showed that the fluorescence signals of A2(35)-DLS1 and A2(35)-DLS2 to target cells were similar to that of A2(35), while the fluorescence signal of the BAD to target cells increased significantly (Figure 3B), suggesting an enhanced binding ability of the bivalent BAD to target cells compared to that of a monovalent aptamer. Moreover, we further evaluated the binding affinity by determining the dissociation constants (K_d_) of A2(35) and the BAD binding to target KYSE410 cells. As shown in Figure 3C, the K_d_ values of A2(35) and the BAD to target cells were in low nanomolar levels, and the K_d_ of the BAD (7.7 ± 2.7 nM) was significantly lower than that of A2(35) (58.1 ± 30.4 nM), indicating that the BAD can bind to target cells with a higher affinity. The above results demonstrated that the constructed bivalent BAD has a stronger binding ability to target cells than a monovalent aptamer.

### 2.5. Construction of the BADD

**Figure 4:**
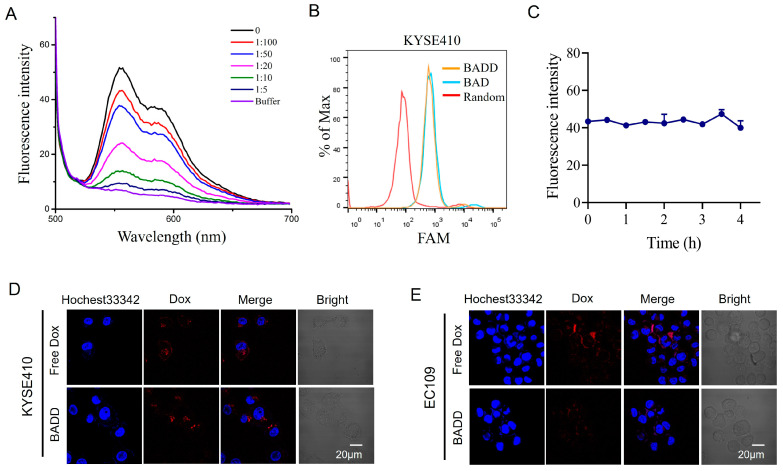
Construction and selective internalization of the BADD. (**A**) Fluorescence spectra of the BADD with different ratios of the BAD to Dox. The Dox concentration was fixed at 10 μM. (**B**) Binding abilities of the BAD and the BADD to target KYSE410 cells analyzed by flow cytometry. 250 nM of the BAD or the BADD was incubated with KYSE410 cells for 30 min. (**C**) Stability analysis of the BADD in a serum-free medium by detecting Dox fluorescence at different times. Internalization of the BADD to target KYSE410 cells (**D**) and control EC109 cells (**E**) were visualized by confocal imaging. 2 μM of the BADD was incubated with the cells for 2 h in a serum-free medium. Dox fluorescence was displayed in red. Cell nuclei were stained with Hochest33342 (blue). The scale bar is 20 μm.

After formation of the BAD, it was used as the DNA carrier to load Dox to construct the BADD for targeted killing of ESCC cells. Because the inherent fluorescence of Dox is quenched when Dox is intercalated in GC base-pairs, the fluorescence spectra of the BADD was recorded to verify the loading of Dox into the BAD. As displayed in Figure 4A, the fluorescence of Dox was gradually decreased with an increasing ratio of the BAD to Dox, indicating that Dox was successfully loaded into the BAD. When the ratio of the BAD to Dox was 1:5, the fluorescence quenching almost reached saturation, indicating that the maximum drug loading was achieved with five Dox loaded into one BAD. Next, we investigated whether the Dox loading affected the binding of the BADD to target KYSE410 cells. The flow cytometry results showed that the BADD had a similar binding ability to target cells compared to the BAD (Figure 4B), suggesting that Dox loading had no reduced effects on the BADD binding to target cells. Additionally, the stability of the BADD in a serum-free medium was evaluated by detecting the fluorescence of leaching Dox. The results showed that the fluorescence intensity did not change significantly within 4 h (Figure 4C), suggesting that there was almost no leaching of free Dox into the medium and the BADD was stable in the medium.

### 2.6. Selective Internalization of the BADD

Subsequently, selective internalization of the BADD into target cells was evaluated using confocal imaging. The confocal images showed obvious Dox fluorescence in target KYSE410 cells, but not in control EC109 cells, after treatment with the BADD (Figure 4D), and showed similar strong Dox fluorescence in both KYSE410 and EC109 cells after treatment with free Dox (Figure 4E). These results indicated that the BADD can be selectively internalized into target cells and release Dox drugs, while free Dox can non-selectively enter into target and control cells and lead to undesired killing of control cells.

### 2.7. BADD-Induced Selective Killing of Target Cells

**Figure 5:**
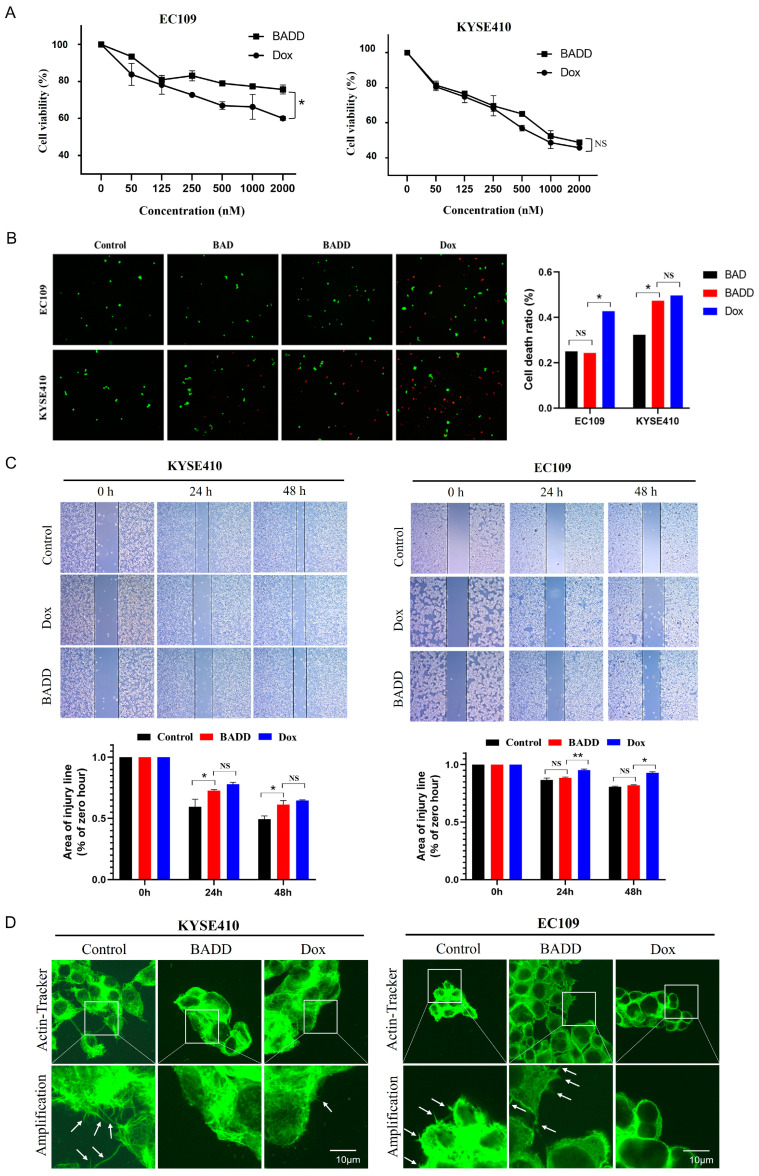
BADD-induced selective killing of target cells. (**A**) CCK-8 analysis of target KYSE410 and control EC109 cells treated with different concentrations (0, 0.05, 0.125, 0.25, 0.5, 1, and 2 μM) of Dox and the BADD for 2 h. (**B**) Cellular activity analysis of KYSE410 and EC109 cells treated with 2 μM of the BAD, Dox, and the BADD for 2 h and stained by Calcein AM/PI. Calcein AM-stained living cells are in green, and PI-stained dead cells are in red. The cell death ratio (number of dead cells/number of all cells) was calculated. (**C**) Wound healing analysis of KYSE410 and EC109 cells treated with 2 μM of Dox and the BADD for 2 h. The ratios of the wound areas at 24 and 48 h to that at 0 h were calculated. The asterisks denote significance (* *p* < 0.05; ** *p* < 0.01), NS denotes no significance. (**D**) Confocal images of lamellipodia at the edge of KYSE410 and EC109 cells treated with 2 μM of Dox and the BADD for 2 h. An actin-Tracker Green-488 dye was used to visualize lamellipodia. The arrow indicates lamellipodia. The scale bar is 10 μm.

To evaluate BADD-induced selective killing of target cells, CCK-8 analysis was performed to detect cell viability. As shown in Figure 5A, the cell viability of control EC109 cells treated with the BADD was significantly higher than that of EC109 cells treated with free Dox, and the cell viabilities of target KYSE410 cells treated with the BADD and free Dox were similar. These results indicated that the BADD can selectively kill target cells, but not control cells. Calcein AM/PI staining was further performed to verify BADD-induced selective killing of target cells. Target KYSE410 and control EC109 cells were treated with the BAD, the BADD, and free Dox, respectively, and the corresponding cell death ratios were calculated. The results (Figure 5B) showed that the cell death ratios of EC109 cells treated with the BAD and the BADD, respectively, were significantly lower than that of EC109 cells treated with free Dox, while the cell death ratio of KYSE410 cells treated with BADD and free Dox were similar, suggesting that Dox can non-selectively kill KYSE410 and EC109 cells while the BADD can selectively kill target KYSE410 cells. Moreover, wound healing analysis was also performed to validate this effect. After the target KYSE410 and control EC109 cells were treated with free Dox and the BADD for 2 h in a serum-free medium and for 24 and 48 h in a complete medium, the corresponding wounded areas were measured. As shown in Figure 5C, the wounded areas of KYSE410 cells treated with free Dox and BADD were similar, while the wounded area of EC109 cells treated with BADD was lower than that of EC109 cells treated with free Dox, indicating that BADD can specifically inhibit target cell growth and migration. Lamellipodia can promote cell migration, which is usually used as the hallmark of migrating cells [22]. Therefore, lamellipodia imaging was performed to verify BADD-induced changes of cell migration ability. As shown in Figure 5D, almost no lamellipodia was observed on the target KYSE410 cells treated with the BADD compared to that on the control, while obvious lamellipodia could be observed on the EC109 cells treated with the BADD, which further verified that the BADD can specifically inhibit target KYSE410 cell growth and migration, but not control EC109 cells. The above results demonstrated that the BADD can selectively inhibit cell growth and may be used as an effective therapeutic reagent for targeted chemotherapy of ESCC.

In previous work, bivalent aptamers are usually used to deliver siRNA for targeted therapy, but it may need complicated design due to integration of siRNA sequences [40,41,42]. Circular bivalent aptamers have also been reported to deliver therapeutics with enhanced stability, while enzyme-catalyzed ligation make the synthesis of circular bivalent aptamers not easy [43,44,45]. Compared to the reported bivalent aptamer complex, the developed BADD can be fast constructed through simple DNA self-assembly and noncovalent intercalation of Dox without difficult sequence design. Because the BADD is composed of the target BAD carrier and Dox, it can specifically enter into cancer cells and release Dox and then kill the cancer cells through Dox. Therefore, the mechanism of the BADD killing ESCC cells is similar to that of Dox killing ESCC cells. As we know, Dox is an FDA-approved drug to treat various cancers, and its mechanism of action for killing tumor cells is well-known. According to the reports, Dox can induce cell death, especially apoptosis, via multiple modes of action, such as intercalate into DNA, inhibit topoisomerase II, produce reactive oxygen species, disrupt mitochondria function and evict histone [8,46,47].

Drug resistance often occurs in chemotherapy. It has been reported that EC109 cells are relatively sensitive to both cisplatin and paclitaxel while KYSE410 cells are relatively resistant to cisplatin [48]. Gene expression profiling is closely associated with these performances; for example, overexpression of MUC4 and MUC20 contributes to drug resistance, while overexpression of MUC13 contributes to drug sensitivity in ESCC cells. The drug resistance of the BADD in ESCC cells remains to be clarified. Although the BADD is feasible to selectively kill cancer cells in vitro, there are some limitations for its in vivo applications. For instance, the BAD carrier is a DNA in nature, so the BADD is probably sensitive to nuclease and may degrade in complex living systems [20]. In the future, improving the biostability of the BADD may be helpful for its in vivo applications. Chemical modifications, such as phosphorothioate, inverted dT, 2′-O-methyl, 2′-fluoro, locked nucleic acid modifications [49,50,51], or integration with DNA nanostructures [52,53], would be available strategies for improving the biostability of the BADD.

## 3. Conclusion

In summary, we have developed the bivalent aptamer-DNA carrier-Dox conjugate (BADD) for targeted killing of ESCC cells. Firstly, a truncated A2(35) aptamer was obtained through optimization of an intact A2(80) aptamer. The flow cytometry results demonstrated that the A2(35) aptamer retained a strong binding ability to target KYSE410 cells. Then, the A2(35) aptamer was used as the recognition molecule to fuse with GC base-rich DNA sequences for constructing the bivalent aptamer-DNA carrier (BAD) through simple DNA hybridization. The agarose gel electrophoresis results showed that the BAD was successfully constructed, and flow cytometry assays showed that the BAD had a stronger binding ability than monovalent A2(35), and the K_d_ of the BAD was significantly lower than that of the monovalent A2(35). The BAD was then used to carry the chemotherapeutic drug Dox to construct the BADD. The fluorescence spectrum analysis showed that Dox was successfully loaded into the BAD and formed the BADD with a drug loading ratio of approximately 1:5. The flow cytometry assays showed that the BADD can bind to target cells strongly, and confocal imaging results further displayed that the BADD can be specifically internalized into target cells and release Dox. CCK-8 assays showed that the BADD can specifically kill target cells, but no significant killing effects on control cells, indicating selective cytotoxicity of the BADD. The selective cytotoxicity was further verified by Calcein AM/PI staining and wound healing analysis. The proposed BADD can be simply constructed and has great binding and targeting ability, which is expected to provide a feasible strategy for targeted chemotherapy of ESCC.

## 4. Materials and Methods

### 4.1. Chemicals and Materials

An RPMI-1640 culture medium and a fetal bovine serum (FBS) were purchased from Biological Industries (Beit-Haemek, Israel). DNA sequences were synthesized and purified by Sangon Biotech (Shanghai, China). Doxorubicin, phosphate-buffered saline (PBS), and a DNA marker were purchased from BBI (Shanghai, China). Agarose was purchased from Invitrogen (Waltham, MA, USA). A GelRed dye was purchased from Biosharp (Hefei, China). CCK-8 was purchased from Topscience (Shanghai, China). A Hoechst 33,342 dye, a Calcein AM/PI dye, bovine serum albumin (BSA), and a DNA loading buffer were purchased from Solarbio (Beijing, China). An Actin-Tracker Green-488 dye was purchased from Beyotime (Shanghai, China). The washing buffer was 0.01 M PBS with 4.5 g/L glucose and 5 mM MgCl_2_. The binding buffer was a washing buffer with 1 mg/mL BSA and 0.1 mg/mL yeast tRNA.

### 4.2. Cells and Culturing

KYSE410 and EC109 ESCC cell lines were obtained from the Department of Pathophysiology, School of Basic Medical Sciences, Zhengzhou University. KYSE410 and EC109 cells were cultured in an RPMI-1640 medium supplied with 10% FBS and 100 μg/mL penicillin-gentamicin. The cells were aseptically cultured at 37 °C in a humidified incubator with 5% CO_2_.

### 4.3. Preparation and Characterization of the BAD and the BADD

To construct the BAD, equal proportions of 1 μM of A2(35)-DLS1 and 1 μM of A2(35)-DLS2 were dissolved in PBS, heated at 95 °C for 5 min and then cooled to room temperature slowly. Agarose gel electrophoresis (2.5%, 100 V, 50 min) was performed to characterize the formation of the BAD. The gel was stained by the GelRed dye and subjected to UV imaging. To construct the BADD, 2 μM of BAD was mixed with 10 μM of Dox in PBS for 30 min at room temperature. To evaluate the Dox loading, the BAD was mixed with 10 μM of Dox in different proportions (BAD:Dox = 0, 1:100, 1:50, 1:20, 1:10, and 1:5). The Dox fluorescence was recorded using a fluorescence spectrometer.

### 4.4. Flow Cytometry Analysis

A2(80), A2(40), A2(35), A2(19), A2(12), A2(35)-DLS1, A2(35)-DLS2, and random sequences were separately labeled with an FAM fluorescence group at the 5’ end for flow cytometry analysis. Generally, KYSE410 or EC109 cells (approximately 1 × 10^5^) were mixed with different probes (250 nM) in a binding buffer for 30 min on ice. After incubation, cells were washed with the washing buffer for 3 times and resuspended in 400 μL of the binding buffer for flow cytometry analysis. The data were processed by Flowjo 7.6 software. To determine the dissociation constants (K_d_) of A2(35) and the BAD to KYSE410 cells, different concentrations (0, 10, 25, 50, 100, 200, and 500 nM) of A2(35) or the BAD were separately mixed with KYSE410 cells, and the fluorescence values of their binding were detected by flow cytometry. K_d_ was calculated by Y = B_max_ X/(K_d_ + X) using GraphPad Prism 8 software, where X represents the probe concentration and Y represents the fluorescence value.

### 4.5. Confocal Imaging

KYSE410 and EC109 cells were seeded in 35 mm confocal dishes (BDD012035, JET BIOFIL, Guangzhou, China) and cultured for 24 h. After the old medium was removed, 2 μM of Dox or the BADD were incubated with the cells for 2 h in a serum-free medium. After washing with PBS for 3 times, the cells were mixed with Hoechst 33,342 for cell nuclei staining. After washing, the medium was added, and the cells were imaged by a laser confocal microscope (Olympus, Tokyo, Japan). Dox fluorescence was excicted at a wavelength of 545 nm and collected at a wavelength of 570 nm with a long-pass filter; Hoechst 33,342 fluorescence was excited at a wavelength of 405 nm and collected at a wavelength range of 435–460 nm. To visualize lamellipodia, KYSE410 and EC109 cells were treated with 2 μM of Dox or the BADD for 2 h. After cultured in the medium for 48 h, the cells were fixed with 3% paraformaldehyde for 15 min and then permeabilized with 0.3% Triton X-100 for 5 min. Afterwards, the cells were stained with the Actin-Tracker Green-488 dye for 1 h and captured by the confocal microscope.

### 4.6. CCK-8 Analysis

KYSE410 and EC109 cells (6000 cells/well) were seeded in 96-well plates and cultured for 24 h. After removing the old medium, different concentrations (0, 0.05, 0.125, 0.25, 0.5, 1, and 2 μM) of Dox or the BADD were incubated with the cells for 2 h in a serum-free medium. Then, the solution was removed and replaced with a culture medium. The cells were then cultured at 37 °C for 48 h in the incubator. Subsequently, 10 μL of CCK-8 solution was added to each well and incubated at 37 °C for 2 h. The absorbance at 450 nm was detected by a microplate reader.

### 4.7. Calcein AM/PI Staining

KYSE410 and EC109 cells (1.5 × 10^4^ cells/well) were seeded into 24-well plates and cultured for 24 h. After removing the old medium, 2 μM of Dox or the BADD was added into each well and cultured for 2 h in a serum-free medium. The solution was removed, and cultivation continued at 37 °C for 48 h in the medium. The adherent cells and the detached cells suspended in the culture medium were all collected. The cells were stained with the diluted Calcein AM/PI solution at 37 °C for 30 min in dark. The cell images were captured by a fluorescence microscope.

### 4.8. Wound Healing Analysis

KYSE410 and EC109 cells (5 × 10^5^ cells/well) were seeded into 6-well plates and cultured at 37 °C for 24 h. A 10 μL pipette tip was used to wound the cells, and the detached cells were washed with PBS. Then, 2 μM of Dox or the BADD was added into each well with a serum-free medium. After incubation for 2 h, the solution was removed, and cultivation continued at 37 °C. Cell images were captured using a microscope at 0, 24, and 48 h from the same field. The wound healing area was measured by ImageJ 2 software.

## Figures and Tables

**Table 1:** Detailed DNA sequences used in the work.^a^

Name	Sequences (5′–3′)
A2(80)	AGAAGGAAGGAGAGCGACACCACCACGCGAATGCTATCGGGGCTAAGTATCAAAATGAGCTATCAGTGGTCGGTCGTCAT
A2(40)	CACCACGCGAATGCTATCGGGGCTAAGTATCAAAATGAGC
A2(35)	CGCGAATGCTATCGGGGCTAAGTATCAAAATGAGC
A2(19)	GCTAAGTATCAAAATGAGC
A2(12)	CGAATGCTATCG
A2(35)-DLS1	CGTCGTCGTCGTCGTCGTTTTCGCGAATGCTATCGGGGCTAAGTATCAAAATGAGC
A2(35)-DLS2	ACGACGACGACGACGACGTTTCGCGAATGCTATCGGGGCTAAGTATCAAAATGAGC

^a^ Aptamer recognition sequences are in brown, and drug loading sequences are in orange.

## Data Availability

The data presented in this study are available upon request from the corresponding author.
